# Supplementary material for: Comparison of loop extrusion and diffusion capture as mitotic chromosome formation pathways in fission yeast
Source: Nucleic Acids Res. 2021 Jan 12;49(3):1294–312. doi: 10.1093/nar/gkaa1270 (PMC7897502; doi:10.1093/nar/gkaa1270)
Supplement: gkaa1270_Supplemental_Files [file gkaa1270_supplemental_files.zip › Supplementary Data.pdf]

Comparison of loop extrusion and diffusion capture as mitotic chromosome formation pathways in fission yeast

Tereza Gerguri, Xiao Fu, Yasutaka Kakui, Bhavin Khatri, Christopher Barrington, Paul A. Bates and Frank Uhlmann

Supplementary Data

Supplementary Figure S1 – S6 2

Supplementary Table S1 8

Supplementary Movie Legends 9

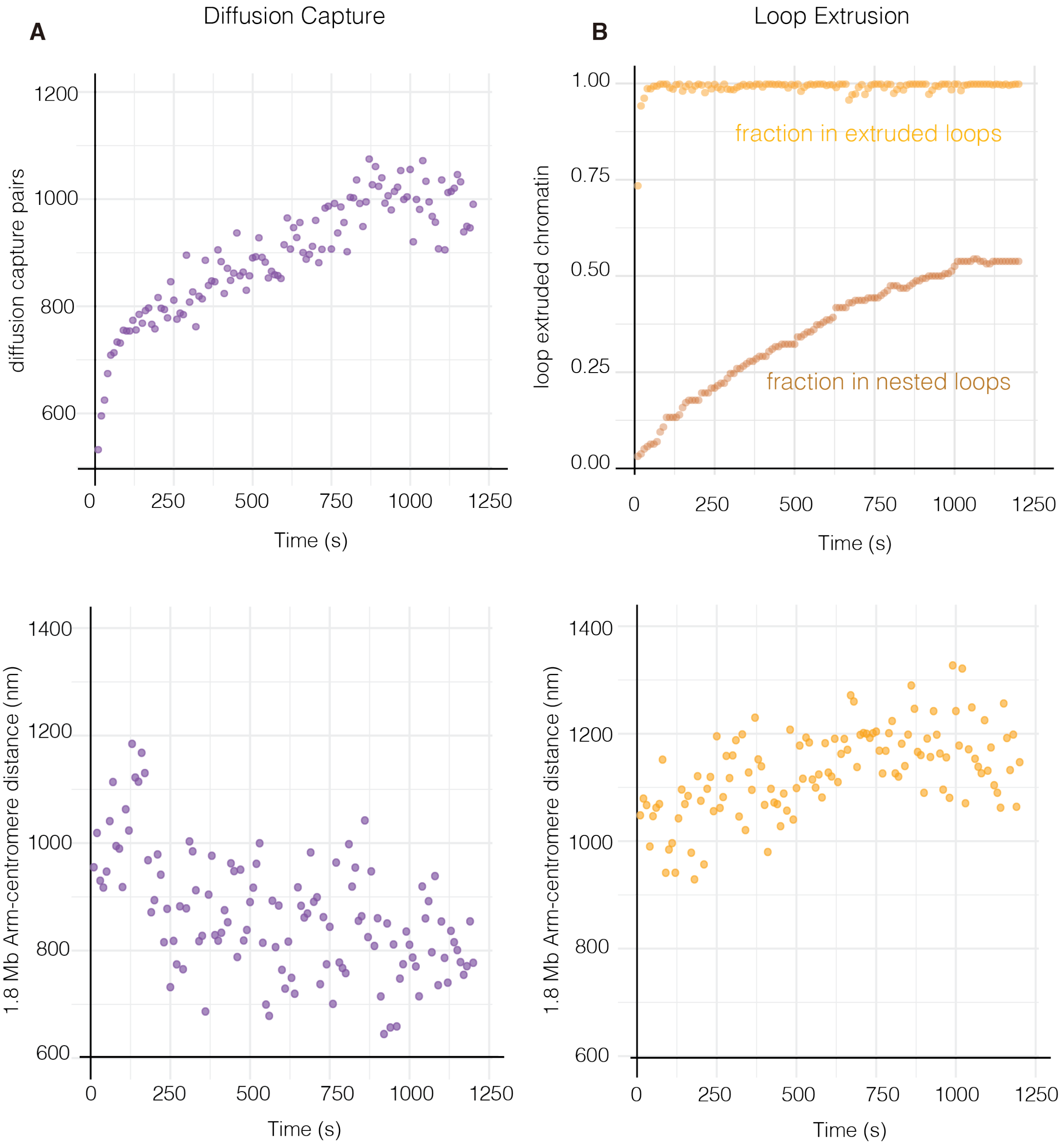

Supplementary Figure 1. Time development of mitotic diffusion capture and loop extrusion simulations. (**A**) Diffusion capture simulations. Starting from a relaxed chromatin chain, the time development of diffusion capture pair formation, as well as the axial chromosome distance of the *in silico* fluorophore pair analyzed in Figure 2, are plotted over time. The medians from the 10 simulation repeats are shown at 10 second intervals. (**B**) Loop extrusion simulations. As in (**A**), but the fraction of the chromatin chain that is contained in loops (orange) or nested loops (brown), as well as the *in silico* fluorophore pair distance, are shown.

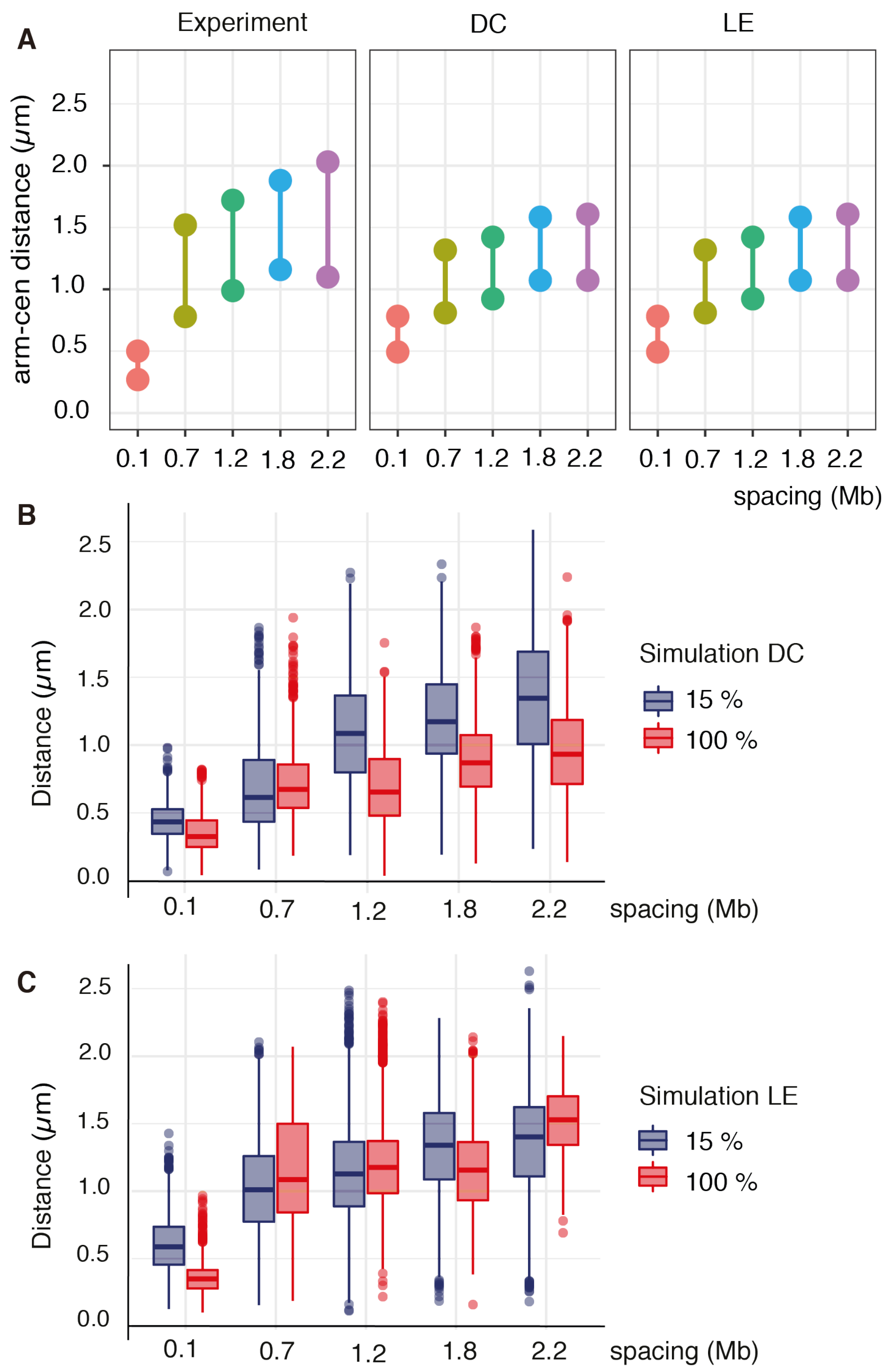

Supplementary Figure 2. Axial chromosome lengths in experimental data and diffusion capture and loop extrusion simulations, measured over different genomic distances. (**A**) Line plots depict minimal and maximal values of distances recorded of fluorophore pairs at the indicated genomic spacing during experimental interphase (left panel, data from (29)), as well as the interquartile ranges from 1,200 measurements at 10 second intervals during 10 simulation repeats during diffusion capture (middle panel) and loop extrusion (right panel) simulations using interphase condensin levels. (**B, C**) Euclidean distance distributions of *in silico* fluorophores at the indicated genomic spacing for diffusion capture (**B**) or loop extrusion simulations (**C**), using interphase (blue, 15% condensin) and mitotic conditions (red, 100% condensin). Boxes depict medians and interquartile ranges.

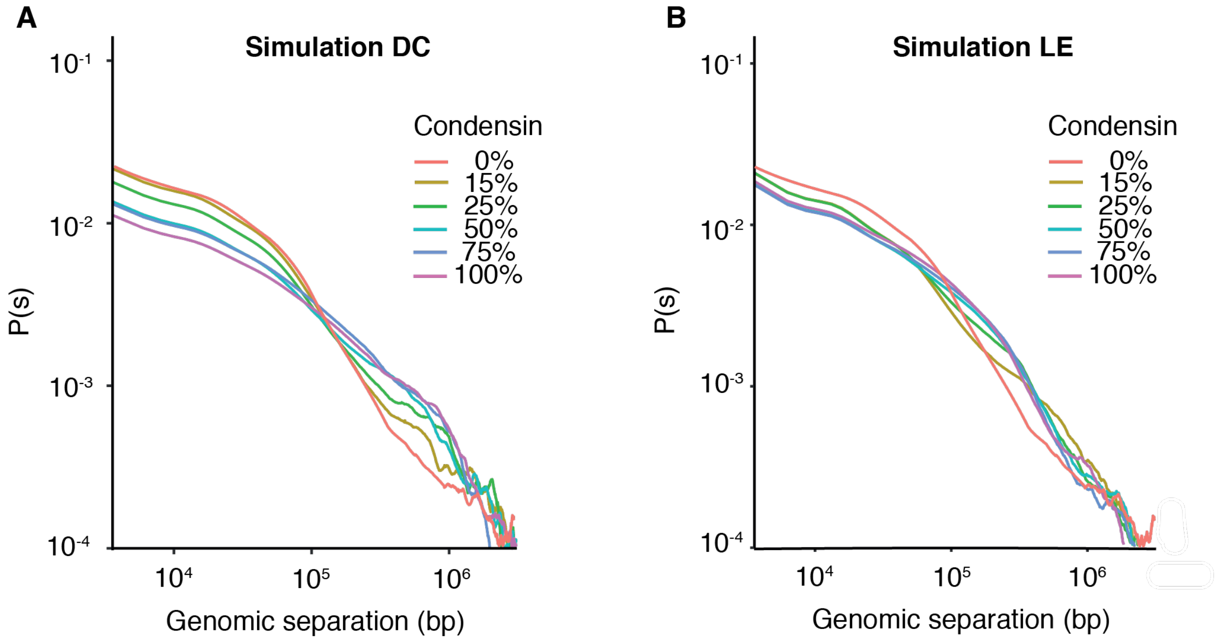

Supplementary Figure 3. Condensin concentration-dependent changes to the contact probability distribution in the diffusion capture and loop extrusion models. (**A, B**) Contact probability as a function of genomic separation, as in Figure 3, is plotted for different condensin concentrations during diffusion capture (**A**) and loop extrusion simulations (**B**). 12,000 conformations, recorded at 1 second intervals during 10 simulation replicates, were analyzed in each case.

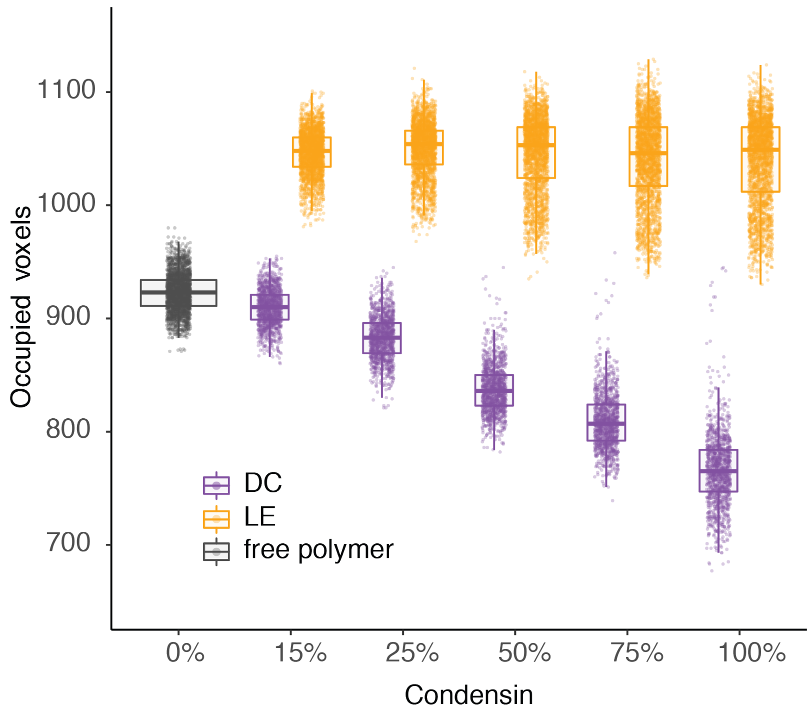

Supplementary Figure 4. Condensin concentration-dependent chromosome volume compaction during simulated diffusion capture and loop extrusion. Occupied voxel distributions during diffusion capture (purple) and loop extrusion (orange) at different condensin concentrations is shown. 1,200 snapshots, taken at 10 second intervals from 10 simulation replicates were analyzed. Boxes indicate the medians and interquartile ranges.

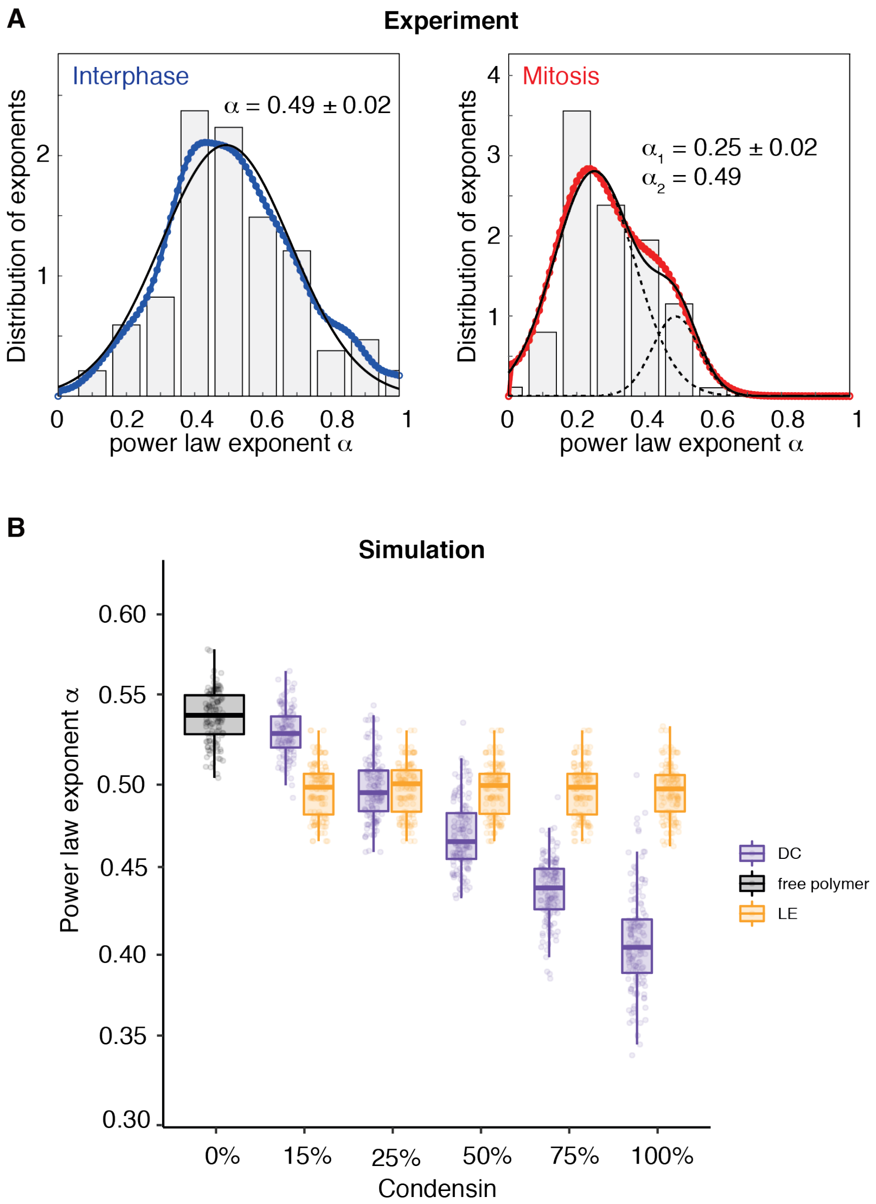

Supplementary Figure 5. Additional analyses of *in vivo* and *in silico* chromatin mobility. (**A**) Histogram (grey bars) and kernel density estimate (colored circles) of probability density of individual MSD exponents in interphase and mitosis. The solid black line represents a single Gaussian fit of the interphase distributions (n = 595). The dashed lines show a double Gaussian fit for the exponent distribution in mitosis (n = 271), where the mean of the second Gaussian is constrained to the mean measured in interphase, assuming that it reflects contaminating interphase cells in the population. (**B**) Condensin concentration-dependence of chromatin chain mobility in the diffusion capture and loop extrusion models. The distribution of MSD exponents of a free chromatin chain (black) is compared to the indicated condensin concentrations during diffusion capture (purple) and loop extrusion simulations (orange). 2 second traces were analyzed every 30th second during 10 simulation repeats. Boxes indicate the medians and interquartile ranges. We previously used similar simulations to arrive at an MSD exponent of a free polymer chain α = 0.57 ± 0.11 (24); here 0.53 ± 0.03 compatible within the confidence intervals of both studies. A difference in approach was the use of a nuclear constraint in our current study, which was not included in our previous simulations and which might have introduced a certain limit on mobility.

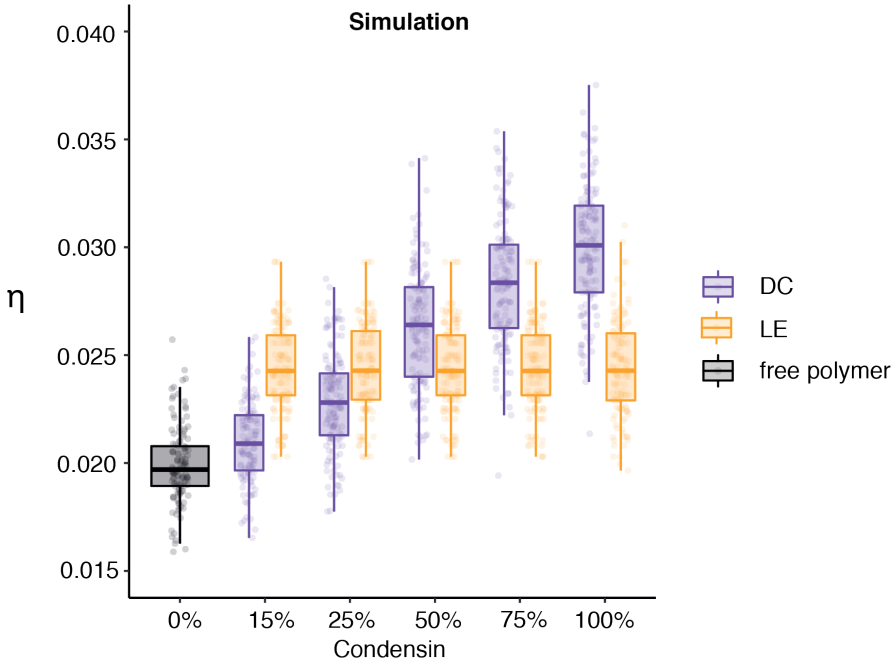

Supplementary Figure 6. Condensin concentration-dependence of anisotropic motion during diffusion capture and loop extrusion. The distribution of anisotropy exponents of a free chromatin chain (black) is compared to the effect of the indicated condensin concentrations during diffusion capture (purple) and loop extrusion simulations (orange) shown in Supplementary Figure S5. Boxes indicate the medians and interquartile ranges.

Supplementary Table S1. Yeast strains used in this study.

YUK760	*h^+ SV40-GFP-atb2-LEU2 P_{nmt41}-kan-slp1 mis6-2mCherry-hph ura4-D18 his2 ade6::ade6$^+$-P_{adh15}-skp1-OsTIR1-bsd-P_{adh15}-skp1-AtTIR1-2NLS*	Fig. 4A,B
YUK765	*h^+ SV40-GFP-atb2-LEU2 nat-P_{nmt81}-cut14-IAA-ura4$^+$ P_{nmt41}-kan-slp1 mis6-2mCherry-hph ura4-D18 his2 ade6::ade6$^+$-P_{adh15}-skp1-OsTIR1-bsd-P_{adh15}-skp1-AtTIR1-2NLS*	Fig. 4B
YUK799	*h- P_{nmt41}-kan-slp1 TetR-tdTom::leu1+ LacI-eGFP::his7+ ChrII-3.6Mb::TetO-hphMX ChrI-1.95Mb::LacO-natMX6 ade6::ade6$^+$-P_{adh15}-skp1-OsTIR1-bsd-P_{adh15}-skp1-AtTIR1-2NLS nat-P_{nmt81}-cut14-IAA-ura4$^+$ ura4.D18*	Fig. 5A and 6A, S5A
YUK808	*h- P_{nmt41}-kan-slp1 TetR-tdTom::leu1+ LacI-eGFP::his7+ ChrII-3.6Mb::TetO-hphMX ChrI-1.95Mb::LacO-natMX6 lys1 ade6-M210*	Fig. 5A and 6A, S5A
YUK1062	*h^+ P_{nmt41}-kan-slp1 cdc2-as-M17-bsd cut14-SNAP-HA-hph ade6::ade6$^+$-P_{adh15}-skp1-OsTIR1-nat-P_{adh15}-skp1-AtTIR1-2NLS ura4.D18 his2*	Fig. 7

Supplementary Movie Legends

Supplementary Movie S1. Interphase diffusion capture simulation. Representative polymer simulation of the 3.76 Mb chromatin chain with condensin performing diffusion capture. 15% of condensin binding sites are occupied to represent *in silico* interphase. The first 100 seconds of the simulation are captured. The grey fiber represents the chromatin chain, condensin binding sites are indicated in light purple, while condensin molecules are shown as pairs of blue spheres. The red bead indicates the in silico fluorophore positioned at 1.95 Mb.

Supplementary Movie S2. Mitotic diffusion capture simulation. As Movie S1, but 100% of condensin binding sites are occupied to represent *in silico* mitosis.

Supplementary Movie S3. Interphase loop extrusion simulation. Representative polymer simulation of the 3.76 Mb chromatin chain with condensin performing loop extrusion. Condensin molecules corresponding to 15% of binding sites are present in the system to represent *in silico* interphase. The first 100 seconds of the simulation are captured. The grey fiber represents the chromatin chain, turning orange when it forms part of an extruded loop. Condensin binding sites are indicated in light purple, while condensin molecules are shown as pairs of blue spheres. The red bead indicates the in silico fluorophore positioned at 1.95 Mb.

Supplementary Movie S4. Mitotic loop extrusion simulation. As Movie S3, but condensin corresponding to 100% of condensin binding sites is present in the system to represent *in silico* mitosis.
